# Epigenetically upregulated oncoprotein PLCE1 drives esophageal carcinoma angiogenesis and proliferation via activating the PI-PLCε-NF-κB signaling pathway and VEGF-C/ Bcl-2 expression

**DOI:** 10.1186/s12943-018-0930-x

**Published:** 2019-01-04

**Authors:** Yunzhao Chen, Dandan Wang, Hao Peng, Xi Chen, Xueping Han, Jie Yu, Wenjie Wang, Lirong Liang, Zheng Liu, Yi Zheng, Jianming Hu, Lan Yang, Jun Li, Hong Zhou, Xiaobin Cui, Feng Li

**Affiliations:** 1 Department of Pathology and Key Laboratory for Xinjiang Endemic and Ethnic Diseases, The First Affiliated Hospital, Shihezi University School of Medicine, Shihezi, 832002 China; 2 Department of Pathology and Medical Research Center, Beijing Chaoyang Hospital, Capital Medical University, Beijing, 100020 China; 3 The people’s hospital of Suzhou National Hi-Tech District, Suzhou, 215010 China; 4 Department of Gastroenterology, The First Affiliated Hospital, Shihezi University School of Medicine, Shihezi, 832002 China; 5 Department of Ultrasound, The First Affiliated Hospital, Shihezi University School of Medicine, Shihezi, 832002 China; 6 Bone Research Program, ANZAC Research Institute, University of Sydney, Sydney, NSW Australia

**Keywords:** Esophageal carcinoma, PLCE1, NF-κB, Angiogenesis, Proliferation

## Abstract

**Background:**

Esophageal squamous cell carcinoma (ESCC) is one of the most lethal malignancies. Neovascularization during tumorigenesis supplies oxygen and nutrients to proliferative tumor cells, and serves as a conduit for migration. Targeting oncogenes involved in angiogenesis is needed to treat organ-confined and locally advanced ESCC. Although the phospholipase C epsilon-1 (PLCE1) gene was originally identified as a susceptibility gene for ESCC, how PLCE1 is involved in ESCC is unclear.

**Methods:**

Matrix-assisted laser desorption ionization time-of-flight mass spectrometry were used to measure the methylation status of the PLCE1 promoter region. To validate the underlying mechanism for PLCE1 in constitutive activation of the NF-κB signaling pathway, we performed studies using in vitro and in vivo assays and samples from 368 formalin-fixed esophageal cancer tissues and 215 normal tissues with IHC using tissue microarrays and the Cancer Genome Atlas dataset.

**Results:**

We report that hypomethylation-associated up-regulation of PLCE1 expression was correlated with tumor angiogenesis and poor prognosis in ESCC cohorts. PLCE1 can activate NF-κB through phosphoinositide-phospholipase C-ε (PI-PLCε) signaling pathway. Furthermore, PLCE1 can bind p65 and IκBα proteins, promoting IκBα-S32 and p65-S536 phosphorylation. Consequently, phosphorylated IκBα promotes nuclear translocation of p50/p65 and p65, as a transcription factor, can bind vascular endothelial growth factor-C and bcl-2 promoters, enhancing angiogenesis and inhibiting apoptosis in vitro. Moreover, xenograft tumors in nude mice proved that PLCE1 can induce angiogenesis, inhibit apoptosis, and increase tumor aggressiveness via the NF-κB signaling pathway in vivo.

**Conclusions:**

Our findings not only provide evidence that hypomethylation-induced PLCE1 confers angiogenesis and proliferation in ESCC by activating PI-PLCε-NF-κB signaling pathway and VEGF-C/Bcl-2 expression, but also suggest that modulation of PLCE1 by epigenetic modification or a selective inhibitor may be a promising therapeutic approach for the treatment of ESCC.

**Electronic supplementary material:**

The online version of this article (10.1186/s12943-018-0930-x) contains supplementary material, which is available to authorized users.

## Background

Esophageal squamous cell carcinoma (ESCC) is one of the most common cancers and the sixth leading cause of cancer-related deaths worldwide [1]. ESCC is characterized by a progressive developmental pattern with a poor prognosis. ESCC undergoes a slow, multi-stage, two-way transformation, and it can have various degrees of inflammation, dysplasia, low-grade intraepithelial neoplasia, and high-grade intraepithelial neoplasia, ultimately developing into cancer. In this process, tumor angiogenesis during tumorigenesis contributes to the aggressiveness and poor prognosis of ESCC. Neovascularization supplies oxygen and nutrients to proliferative tumor cells, and serves as a conduit for migration. Thus, understanding molecular mechanisms that contribute to angiogenesis and resistance to apoptosis may help identify the biological basis of ESCC and improve therapy [2, 3].

Phospholipase C epsilon 1 (PLCE1) was identified as a member of the phospholipase family, which is essential for intracellular signaling by catalyzing hydrolysis of a membrane phospholipid, i.e., phosphatidylinositol-4,5-bisphosphate, to generate two important secondary messengers, i.e., diacylglycerol and inositol 1,4,5-trisphosphate [4–6]. PLCE1 was identified to mediate diverse external signals and has been reported to correlate with tumor clinical stages and survival, including hepatocellular carcinoma, colorectal, bladder, gastric, head and neck, and gallbladder cancers [2, 7, 8]. Recent genome-wide association studies (GWAS) indicated that single-nucleotide polymorphisms (SNPs) in PLCE1 can affect gene expression, protein functions, and risk for ESCC [4]. Similarly, we demonstrated that SNPs (rs12263737 and rs2274223) in PLCE1 are associated with esophageal cancer via promoting gene expression in a Chinese-Kazakh population, and the heterozygote of PLCE1 rs2274223 increases susceptibility to human papillomavirus infection in Kazakh patients with ESCC [6, 9]. Our previous investigation also showed that PLCE1 mRNA and protein expression significantly increased in Kazakh ESCC, and that the overexpression of PLCE1 was correlated to poor metastasis and biological aggressiveness [10]. Targeting oncogenic PLCE1 by miR-145 can impair tumor proliferation and metastasis of esophageal cancer [2]. Zhai’s study showed that using CRISPR/Cas9 genome editing technology to knockdown PLCE1, cell migration, and invasion were significantly inhibited by decreasing transcriptional activity of snail in ESCC [11]. Pathophysiological roles of PLCE1 have been studied in various animal models carrying artificial or spontaneous mutations in their chromosomal genes. PLCE1 knockout mice are resistant to 9,10-dimethylbenzanthracene/phorbol 12-myristate 13-acetate-induced two-stage skin chemical carcinogenesis and to intestinal tumorigenesis, which are caused by loss-of-function mutations of adenomatous polyposis coli tumor suppressor gene [12, 13]. In contrast, the overexpression of PLCE1 in the epidermis results in inflammation, which is similar to human psoriasis [14]. Angiogenesis is a regulated process integral to many physiological and pathological situations, including carcinogenesis and tumor growth. The majority of the angiogenic processes are related to inflammation. Modulating the interaction between inflammation and angiogenesis could be an important target for cancer treatment [15]. PLCE1 is key to various inflammations, but there are no reports to describe molecular mechanisms underlying PLCE1 in ESCC angiogenesis.

The nuclear factor-κB (NF-κB) pathway is primarily an inflammatory oncogenic signaling pathway that contributes to angiogenesis and proliferation and is constitutively activated in various human cancers [16–19]. In the resting state, NF-κB p50/p65 binds to its inhibitor protein IκBs and is retained in the form of heterodimers in the cytoplasm. After extracellular signal stimulation, IκBs are phosphorylated by the IkB kinase (IKK) complex, resulting in the degradation of proteasomal IκBs and nuclear translocation of cytoplasmic NF-κB p50/p65. These events activate transcription downstream genes, such as Bcl-2, MMP, FLIP, and VEGF-C, which protect against apoptosis and promote angiogenesis. NF-κB activation is vital to the development and progression of ESCC [20]. NF-κB pathway blockade can sensitize ESCC to chemotherapeutic drugs, inhibit ESCC proliferation, and suppress angiogenesis and metastasis in ESCC [21]. Our previous work showed that PLCE1 expression was positively correlated with NF-κB-related protein in Kazakh patients with ESCC [22]. Du’s paper discussed PLCE1 promotion of renal cell carcinoma growth via the NF-κB-mediated upregulation of VEGF [23]. A similar mechanism has been shown to occur in colon epithelial cells, wherein PLCE1 can activate the NF-κB pathway via PKD-PEA15-RSK to facilitate inflammation and inflammation-associated carcinogenesis [24]. Therefore, we must identify the molecular mechanisms of angiogenesis by which NF-κB signaling is activated in ESCC.

Here, we confirmed that hypomethylation of PLCE1 promoter significantly up-regulated PLCE1 protien expression and the overexpressed PLCE1 was correlated with tumor angiogenesis and poor overall survival for ESCC patients. In in vitro studies, we demonstrated that PLCE1 can activate NF-κB through phosphoinositide-phospholipase C-ε (PI-PLCε) signaling pathway and directly bind p65 and IκBα proteins, thus promoting their phosphorylation. Consequently, phosphorylated IκBα promoted nuclear translocation of p50/p65. As a transcription factor, p65 can directly bind VEGF-C and bcl-2 promoters, enhancing angiogenesis and inhibiting apoptosis. In contrast, the inhibition of PLCE1 induces apoptosis and inhibits angiogenesis of ESCC cells. Xenograft tumors in nude mice also suggest that PLCE1 can induce angiogenesis, inhibit apoptosis, and increase aggressiveness via the NF-κB signaling pathway in vivo. Thus, PLCE1 may regulate the NF-κB signaling pathway and modulation of PLCE1 may provide a therapeutic approach for treating ESCC.

## Methods

### Antibodies and reagents

Sources of antibodies against the following proteins were as follows: PLCE1 (Santa-sc-28,404, 1:200 for WB), PLCE1 (Sigma-Aldrich - HPA015598, 1:50 for IHC and IF), p65 (Abcam-Ab32536, 1:3200 for IHC and 1:10,000 for WB), Phospho-p65 (Abcam-Ab86299, 1:5000 for WB), IκBa (Abcam-Ab32518, 1:200 for IHC and 1:5000 for WB), Phospho-IκBa(Ser32) (CellSignalingTechnology-2859, 1:1000 for WB), IKKα (Abcam-Ab32041, 1:100 for IHC and 1:5000 for WB), phospho-IKKα/β(Ser176/180) (CellSignalingTechnology-2697, 1:1000 for WB), PKCα(Santa-sc-208, 1:1000 for WB), Bax (Abcam-Ab32503, 1:1000 for WB), caspase 3 (Proteintech-19,677, 1:1000 for WB), caspase 7 (Bioss-BAO088–1, 1:1000 for WB), cleaved PARP (Abcam-Ab32561, 1:10,000 for WB), vimentin (Proteintech-60,330, 1:1000 for WB), E-cadherin (Santa-sc-71,009, 1:200 for WB), VEGF-C (Proteintech-22,601-1-AP, 1:600 for WB), Bcl-2 (Beyotime- AB112, 1:1000 for WB), CD34 (ZSGB- ZM0046, 1:200 for IHC), Ki-67 (ZSGB- ZA0502, 1:200 for IHC and IF), and β-actin (Solarbio- RG000120, 1:1000 for WB). U73122 (112648–68-7) was purchased from MedChem Express. Bay11–7082 was purchased from Merck.

### Patients and tissue specimens

A total of 368 ESCC and 215 non-cancerous tissue samples were collected from patients and confirmed by pathological diagnosis. Aside from diagnostic biopsies, chemotherapy, or radiation therapy, no patient underwent previous surgery at the First University Hospital, Shihezi University School of Medicine, the People’s Hospital of Xinjiang Uyghur Autonomous Region, and the Xinjiang Yili Prefecture Friendship Hospital between 1984 and 2014. Tumor-node-metastasis stages of pathological diagnoses for all of the cases were evaluated based on the Cancer Stage Manual (7th Edition; issued in 2009 by the American Joint Committee on Cancer/Union for International Cancer Control). For the 368 ESCC specimens, 215 adjacent non-malignant tissues were controls. All of the patients were enrolled with written informed consent, and the study was approved by the Institutional Ethical Review Board at the First University Hospital, the Shihezi University School of Medicine, and Shihezi University.

### Quantitative analysis of PLCE1 DNA methylation by MALDI-TOF MS

**Fig. 1:**
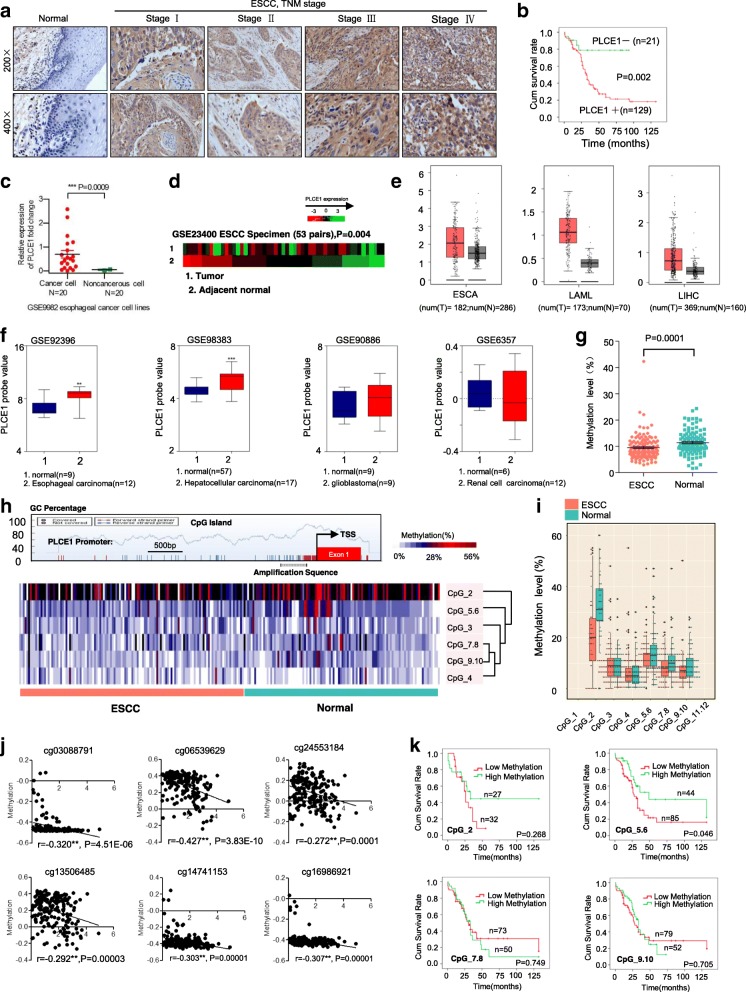
PLCE1 is upregulated in ESCC through aberrant promoter hypomethylation. **a** IHC staining of PLCE1 expression in human ESCC (clinical stages I–IV) and normal esophageal tissues. **b** Kaplan-Meier overall survival curves for patients with ESCC stratified by low (*n* = 21) and high (*n* = 129) expressions of PLCE1 (*P* = 0.002). **c** GEO data (GSE9982) analysis of PLCE1 mRNA expression in normal esophageal cell lines (*n* = 20) and esophageal cancer cell lines (n = 20). ***P < 0.0009, unpaired two-tailed Student’s t-test. **d** Analysis of PLCE1 gene expression in tumor and adjacent non-tumor tissues in NCBI/GEO/GSE23400 (*P* = 0.004). **e** GEPIA analysis of PLCE1 expression in cancerous and normal tissues. ESCA: esophageal carcinoma; LAML: Acute Myeloid Leukemia; LIHC: Liver hepatocellular carcinoma; *P < 0.05. **f** GEO data analysis for the expression of PLCE1 by two-tailed t test (*P < 0.05, **P < 0.01, ***P < 0.001). **g** Genomic structure of PLCE1 CpG dinucleotides over TSS and hierarchical cluster analysis of CpG units’ methylation profiles of PLCE1 promoter region in ESCC (*n* = 132) and normal (*n* = 104). Each vertex indicates one CpG site. Each column represents one sample. Rows are clustering of CpG units, which are single CpG sites or a combination of CpG sites. Color gradient between white and red indicates methylation of each PLCE1 CpG unit in each sample (0–56%). Black represents inadequate or missing data. **h** Comparison of average methylation of PLCE1 promoter of ESCC and normal subjects. The overall methylation level of the target fragment of the PLCE1 promoter was statistically lower (0.0957 ± 0.0456) in ESCC than that in normal tissues (0.1144 ± 0.0464, *P* = 0.0001). **i** Box plot of 6 CpG units in PLCE1 promoter between ESCC and normal tissues. Red and blue spots represent methylation status of one CpG site in ESCC and normal tissues. Dark spots are outliers. In addition to CpG_3 an d CpG_4, the mean methylation levels at CpG_2, CpG_5.6, CpG_7.8, and CpG_9.10 were significantly lower in patients with ESCC (mean methylation = 20.25, 11.84, 8.07, 7.20%, respectively) than those in the controls (mean methylation = 32.38, 13.89, 10.52, 9.37%, respectively; all the *P* < 0.05), *P < 0.05, **P < 0.01, ***P < 0.001 (Mann–Whitney U-test). **j** The methylation status of 6 CpG site was all negatively correlated with PLCE1 expression in TCGA Illumina 450 k infinium methylation beadchip. **k** Kaplan-Meier analysis of survival time according to PLCE1 CpG methylation in ESCC patients

In this assay, 236 samples, namely, 132 ESCC and 104 normal tissue samples, were used for PLCE1 methylation detection. The age was 60.36 ± 8.27 (mean ± SD) years for the cancer samples and 60.94 ± 8.61 years for the normal sample (*P* = 0.60). A total of 85 (64.4%) males and 47 (35.6%) females were selected for the case group and 68 (65.4%) males and 36 (34.6%) females were selected for the control group (*P* = 0.87). DNA was isolated from tissues using DNA Extraction Kit (Qiagen Inc., 56404). NanoDrop spectrophotometer (NanoDrop Technologies Inc.) and gel electrophoresis were used to ensure DNA purity and quality. Purified genomic bisulfite-converted DNA samples were successfully tested by PCR with human PLCE1 primers 5′-aggaagagagGTTGGGTATATTGATGGGGTTTAAT-3′ (forward) and 5′-cagtaatac-gactcactatagggagaaggctACCCCTAAAAACCATCCTTTCTAAC-3′ (reverse). Bisulfite was used to treat genomic DNA by using the EZ DNA Methylation Kit™ in accordance with the manufacturer’s protocol (Zymo Research, D5008). NCBI and CpG island prediction (http://www.ebi.ac.uk/Tools/seqstats/emboss_cpgplot/) were used to identify the sequence of the CpG islands. The analyzed region and CpG sites of the PLCE1 promoter are shown in Fig. 1h. Primer sets for the methylation analysis of the PLCE1 promoter were designed using EpiDesigner (http://epidesigner.com; Additional file 1: Table S1). Mass spectra were collected by MassARRAY Compact MALDI-TOF MS, and the methylation proportions of individual units were generated by EpiTyper 1.0.5 (SEQUENOM). Methylation level was expressed as the percentage of methylated cytosines over the total number of methylated and unmethylated cytosines. The methylation level of each sample was evaluated according to the average methylation values of all CpGs uinits within PLCE1 pomoter. We used the average methylation values 9.6% as a cutoff value to divide all ESCC samples into hypermethylation group and hypomethylation group.

### Cell culture

ESCC cell lines (EC9706 and Eca109) were purchased from the Institute of Biochemistry and Cell Biology of the Chinese Academy of Sciences (Shanghai, China). Cell lines were cultured in Dulbecco’s Modified Eagle’s Medium (Gibco, C11995500) supplemented with 10% fetal bovine serum (Tian Hang Biotechnologies, 0005), 100 U/ml penicillin, and 100 μg/ml streptomycin. HUVECs were isolated from the umbilical cord (from the First University Hospital, Shihezi University School of Medicine; obstetrics, healthy, maternity, and postpartum) and were cultured in Endothelial Cell Medium (Sciencell, 1001). All cell lines were cultured at 37 °C with 5% CO_2_.

### Transfections

LV-R-PLCE1-RNAi (sh-PLCE1) and relative negative scramble control CON053 (sh-sc) lentiviral plasmids were purchased from GeneChem. In most experiments, cells were treated with sh-PLCE1 at MOI of 15. Cell transfections were performed with polybrene (GeneChem, REVG0001) and enhanced infection solution (ENi.S.; GeneChem, REVG0002), HiPerFect transfection reagent (Qiagen, 301,705), or Lipofectamine 2000 reagent (Invitrogen, 11,668–027) by following manufacturers’ instructions. U73122 was also used to inhibit PLCE1, and our results indicated that U73122 exerted time-dependent and dose-dependent inhibition effects on PLCE1 in human esophageal cancer cells. Finally, we used 10 μM for 48 h in follow-up experiments.

### Immunohistochemistry (IHC)

Paraffin-embedded materials were sampled from 368 formalin-fixed esophageal cancer tissues and 215 normal tissues. Samples with 0.6 mm-diameter tissue cores were obtained with a tissue arrayer (ALPHELYS). Tumor samples were fixed with 10% formalin in phosphate-buffered saline (PBS). Paraffin-embedded 4 μm sections were baked at 65 °C for 60 min before rehydration with graded alcohols. Each 4 μm tissue section was deparaffinized and rehydrated. Sections were autoclaved in ethylenediaminetetraacetic acid (EDTA) buffer (pH 9.0) at 130 °C for 10 min in a microwave oven, cooled to 30 °C for 40 min, and incubated with fresh 3% H_2_O_2_ in methanol for 10 min at room temperature. Tissue sections were incubated at 4 °C overnight with antibodies. Negative controls were prepared by replacing primary antibodies with PBS. Tissues were washed thrice in PBS for 5 min before incubation with respective secondary antibody for 30 min at 37 °C. Subsequently, 3,3-diaminobenzidine was used for visualization. Tissue sections were counterstained with hematoxylin. The location of CD34 staining was vascular endothelial cells. Thus, the IHC result of CD34 staining is represented by the MVD. The average of the number of positive vascular endothelial cells was calculated as described previously [25].

### Immunofluorescence (IF) procedure

Cells on glass coverslips (BD) were fixed with 2% paraformaldehyde and permeabilized with 0.2% Triton X-100 in PBS. Samples were then blocked in 5% goat serum in the presence of 0.1% Triton X-100 and stained with the appropriate fluorescence-coupled primary and secondary antibodies.

### Western blot analysis

Homogenized tissues or cells were lysed at 4 °C in radioimmunoprecipitation assay buffer (Solarbio, R0010) mixed with protease and phosphatase inhibitors. Protein lysates were resolved by sodium dodecyl sulfate polyacrylamide gel electrophoresis (SDS–PAGE) and transferred to Immun-Blot PVDF membranes (Solarbio). Equal amounts of protein were boiled, resolved by SDS-PAGE, and transferred to polyvinylidene difluoride membranes. Membranes were incubated in blocking buffer (5% milk, 0.1% Tween20 in Tris-buffered saline) for 1 h and probed overnight with primary antibody at 4 °C. Blots were rinsed thrice (0.1% Tween20 in Tris-buffered saline, 5 min each), followed by incubation with peroxidase-conjugated secondary antibody (Solarbio) for 2 h at room temperature. Proteins were detected using Pierce ECL Plus Western blotting substrate kit (ThermoFisher Scientific).

### Immunoprecipitation (IP) experiments

Cell lysates were prepared by incubating cells in NETN buffer (50 mM Tris-HCl, pH 8.0, 150 mM NaCl, 0.2% Nonidet P-40, 2 mM EDTA) in the presence of Protease Inhibitor Cocktails (Roche) for 20 min at 4 °C. This step was followed by centrifugation at 14,000×g for 15 min at 4 °C. For IP, B500 mg of protein was incubated with control or specific antibodies (1–2 mg) for 12 h at 4 °C with constant rotation. A total of 50 ml of 50% protein G magnetic beads (Invitrogen) were then added, and incubation was continued for an additional 2 h. Beads were then washed five times using lysis buffer. Between washes, beads were collected by magnetic stand (Invitrogen) at 4 °C. Precipitated proteins were eluted from beads by re-suspending beads in 2 SDS-PAGE loading buffer and boiling for 5 min. Boiled immune complexes were subjected to SDS-PAGE, followed by immunoblot with the appropriate antibodies.

### ChIP–quantitative real-time PCR analysis (qPCR)

For ChIP-qPCR assays, cells were treated for 10 min with 5 mM dimethyl 3,30-dithiobispropionimidate-HCl (Pierce) in PBS at room temperature, rinsed with 100 mM Tris–HCl, 150 mM NaCl (pH 8.0), and crosslinked with 1% formaldehyde in PBS at 37 °C for 10 min. Total cell lysates were sonicated to generate 200–500 bp DNA fragments. All resulting precipitated DNA samples were quantified by qPCR. Data are expressed as percentage of input DNA.

### Luciferase assay

Cells (3 × 10^4^) were seeded in triplicates in 48-well plates and allowed to settle for 24 h. A total of 0.1 μg of pNF-κBluc plasmid; or control-luciferase plasmid plus 1 ng of pRL-TK Renilla plasmid (Promega) were transfected into ESCC cells using Lipofectamine 2000 reagent (Invitrogen). After 48 h of transfection, luciferase and Renilla activities were measured using the Dual Luciferase Reporter Assay Kit (Promega).

### MTT assay

For MTT assay, cells were seeded into 96-well plates at 4000 cells per well. After transfection and cell culture for different time periods, 20 μl of MTT (5 mg/ml) (Solarbio, M8180) was added to each well, and cells were incubated for an additional 4 h in the dark. Finally, 125 μl of DMSO (Solarbio, D8370) was added to each well, and optical density was measured at a wavelength of 490 nm.

### Colony formation assay

For colony formation assay, after transfection, 1000 viable cells were plated in six-well plates in triplicate and maintained in complete medium for 15 days. Foci were fixed with 4% polyoxymethylene (Solarbio, P8430) and stained with 0.1% crystal violet (Solarbio, G1061).

### Flow cytometry for detection of apoptosis

To analyze apoptosis rate, 5 × 10^4^ cells were cultured in 24-well plates. After transfection for 48 h, cells were stained with AV/propidium iodide (PI) staining kits (Lianke, AP101–100-kit) and analyzed with a flow cytometer.

### Migration assay

HUVECs (2 × 10^4^) were plated in the upper chamber of BioCoatTM Invasion Chambers (BD, Bedford, MA) and incubated with conditional medium collected from ESCC cells infected with shR-PLCE1, U73122, and Bay11–7082 at 37 °C for 22 h, followed by removal of cells inside the upper chamber with cotton swabs. Migratory cells on the lower membrane surface were fixed in 1% paraformaldehyde, stained with hematoxylin, and counted (ten random × 200 fields per well). Cell counts are expressed as the mean number of cells per field of view. Three independent experiments were performed, and data are presented as mean ± standard deviation (SD).

### Assessment of mitochondria dysfunction by JC-1 staining

JC-1 is a red fluorescent dye which forms aggregates in normal mitochondria. However, JC-1 monomers emit green fluorescence in cells with mitochondrial dysfunction. Cells were seeded in 24-well plates and stimulated as indicated. After treatment, medium was discarded and replaced with 500 μL fresh medium containing 5 μg/mL JC-1. After incubating for 1 h, cells were washed twice with PBS and photographed at 100× magnification under a fluorescent microscope.

### HUVEC tube formation assay

HUVEC tube formation assay was performed. Briefly, 200 μl of pre-cooled Matrigel (Collaborative Biomedical Products) was transferred to each well of a 24-well plate and polymerized for 30 min at 37 °C. HUVECs (2 × 10^4^) in 200 μl of conditioned medium were added to each well and incubated at 37 °C and 5% CO_2_ for 20 h. Capillary tube structure was photographed under a × 100 bright-field microscope and quantified by measuring the total length of completed tubes. Each condition was assessed in triplicate.

### In vivo xenograft mouse model

Animal studies were conducted according to guidelines approved by the University of Shihezi Institutional Animal Care and Use Committee. Athymic nude mice were maintained in specific pathogen-free conditions. Animals were randomly distributed into groups. To induce tumor formation, athymic nude mice were subcutaneously infected with 2 × 10^6^ Eca109 cells into the left axillary area. Eca109 cells were steadily infected with pSIH1-H1-copGFP/shR-PLCE1 or pSIH1-H1-copGFP lentivirus after selection of puromycin. After treatment via injection, tumor length and width measurements were obtained with calipers. Tumor volumes were calculated with the following formula: tumor volume (mm^3^) = (major axial diameter × minor axial diameter 2 × 0.5) thrice weekly. On day 35, tumors were detected by an IVIS imaging system (Caliper Life Sciences, USA), and animals were sacrificed and photographed. Tumors were excised, weighed, photographed, and snap-frozen in liquid nitrogen or formalin-fixed and paraffin-embedded. Paraffin embedded tumor tissues underwent routine histological processing with hematoxylin and eosin (H&E) stain. Cell proliferation in tumors was detected by staining histological sections with a monoclonal antibody against Ki-67 (Zhongshan Biotechnology, ZM-0166); apoptosis was assessed by TUNEL assay (Beyotime, C1086).

### Enzyme-linked immunosorbent assay

IP3 expression was analyzed in cell-free extracts using enzyme-linked immunosorbent assay kits (Elabscience). DAG expression was assessed using enzyme-linked immunosorbent assay kits (Jianglai Shanghai, China). We performed the assay according to the manufacturer’s instructions. Cells were collected using trypsin and resuspended with PBS after treatment with shR-PLCE1, BIM (40 μM) and TPA (150 ng/ml) for 72 h, 24 h, and 36 h, respectively. Then, the cells were subjected to three rounds of repeated freezing and thawing and further broken by ultrasonication.

### Fluorescence confocal microscopy

After the same treatment with ELISA, cells were incubated with 5 μM calcium fluorescence probe Fluo-3 AM (Solarbio) as above using D-Hanks solution. After washing, the fluorescence intensity of calcium was visualized on the scanning confocal microscope under a 63 × oil immersion objective at 490 nm wave. Images were subjected to semi-quantitative analysis using Image J software.

### Statistical analysis

Data were assessed using SPSS (version 17.0) statistical software package (SPSS Inc., Chicago, IL, USA). R Programming Language and GraphPad Prism 5.0 (GraphPad Software Inc., San Diego, CA) were used to describe data. A Mann-Whitney U test was used to compare methylation between groups. Bivariate correlation was used to evaluate correlations. Survival curves were plotted using a Kaplan-Meier method and compared with a log-rank test. Differences were statistically significant at *P* < 0.05 (2-sided). All data are means ± SD.

## Results

### Hypomethylation-associated upregulation of PLCE1 expression reveales poor prognosis of ESCC

The relationship between PLCE1 levels and clinical features of ESCC was further examined in a test cohort with 150 paraffin-embedded, archived ESCC tissues. The end date of follow-up was the date of final contact or the date of death through July 2017. The mean follow-up period is 34.2 months, ranging from 0.5 months to 132 months. Correlation analysis of the cohort indicated that the positive expression of PLCE1 in ESCC was significantly associated with more aggressive tumor phenotypes. As shown in Fig. 1a, PLCE1 expression increased with advanced clinical stage in ESCC, whereas PLCE1 was only found at low levels in normal esophageal tissues. Kaplan-Meier analysis established that in the cohort, 27 months was the median disease-specific survival time for patients with high expressing PLCE1 compared with the 34 months for patients with low expressing PLCE1 (*P* = 0.002, log-rank test; Fig. 1b). As shown in Fig. 1c, the expression level of PLCE1 in GSE9982 esophageal cancer cell lines suggests high expression of PLCE1 in cancer cell lines (*P* = 0.0009; Fig. 1c). Analysis of 53 pairs of ESCC tissue specimens in the GSE23400 database revealed a high expression of PLCE1 in patients with ESCC in the cohort (*P* = 0.004; Fig. 1d). Altogether, these investigations indicate that PLCE1 may be a predictive biomarker for disease outcome in ESCC. Moreover, the GEPIA database in Fig. 1e show that compared to normal, PLCE1 significantly increased expression levels in ESCA, LAML, LIHC patients. Data from the Oncomine database (https://www.oncomine.com/) also confirmed that PLCE1 expression was increased in esophageal carcinoma, hepatocellular carcinoma, glioblastoma and renal cell carcinoma in the four published datasets, GSE92396, GSE98383, GSE90886, and GSE6357 (Fig. 1f). These findings revealed an oncogenic role of PLCE1 in different cancers including ESCC.

Importantly, CpGs residing within PLCE1 were strongly hypomethylated in tumor tissues compared to adjacent normal tissues (Fig. 1g, h). In addition to CpG_3 an d CpG_4, the mean methylation levels at CpG_2, CpG_5.6, CpG_7.8, and CpG_9.10 were significantly lower in patients with ESCC than those in the controls (Fig. 1i and Additional file 1: Table S2). Notably, a significant inverse correlation was observed for CpG_2 and CpG_5.6 methylation and PLCE1 protein expression (Additional file 1: Table S3). A negative relationship between global PLCE1 methylation and protein expression was also observed (Additional file 1: Table S4). In TCGA Illumina 450 k infinium methylation beadchip, we found that the methylation status of 6 CpG site (cg03088791; cg06539629; cg24553184; cg13506485; cg14741153; cg16986921) was all negatively correlated with PLCE1 expression (Fig. 1j). Furthermore, Kaplan-Meier analysis showed that esophageal carcinoma patients with CpG_5.6 hypomethylation have significantly shorter overall survival than those with relative hypermethylation (log rank *P* = 0.046) (Fig. 1k).

### Overexpression of PLCE1 is relevant with the angiogenesis and proliferation of ESCC

**Fig. 2:**
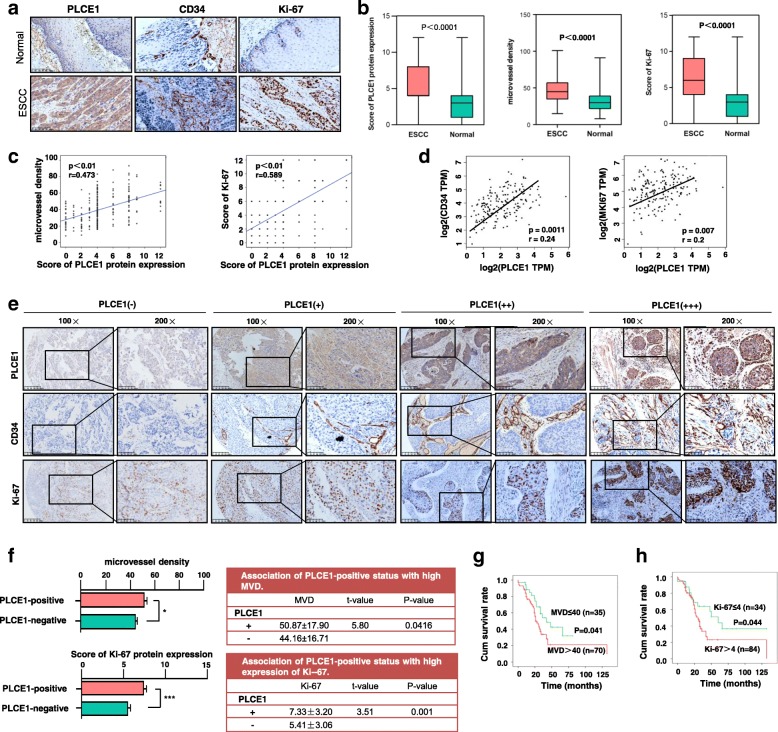
Overexpression of PLCE1 is relevant with the angiogenesis and proliferation of ESCC. **a** Representative IHC images of PLCE1, CD34 and Ki-67 staining (200×) in ESCC and normal esophageal tissue specimens. **b** The score of PLCE1 and Ki-67 and the MVD in ESCC were higher than normal esophageal tissues. Each bar represents the mean ± SD for triplicate experiments. **c, d** The correlation analysis between PLCE1 with MVD and Ki-67 were carried out. PLCE1 and MVD are positively correlated, so as PLCE1 and Ki-67. **e** Representative IHC images of PLCE1, CD34 and Ki-67 staining according to intensities of PLCE1, CD34 and Ki-67 staining in consecutive ESCC tissues. **f** MVD numbers and Ki-67 score were higher in IHC with positive PLCE1 expression compared to negative c-kit expression. **g**, **h** Kaplan-Meier curves for patients. High-MVD (MVD > 40) group (*n* = 70) and Ki-67-positive (*n* = 84) had poor prognosis compared with low-MVD expression group (*n* = 35) and a Ki-67-negative group (*n* = 34)

Angiogenesis is the basic and important stage in the process of ESCC tumorigenesis. Microvessel density (MVD) is the most recognized indicator to evaluate angiogenesis of solid tumors. CD34 is used to label MVD. As PLCE1 has been investigated to be an important predictive marker for ESCC, researchers have worked to clarify the relationship between tumor neovascularization and PLCE1 in ESCC specimens. The PLCE1 and Ki-67-positive expression and MVD, which was labeled by CD34, were significantly higher in the ESCC tissues than in normal esophageal tissues (Fig. 2a, b). Figure 2c, d show the results of the correlation analysis between PLCE1 with MVD and Ki-67. PLCE1 and MVD are positively correlated, as are PLCE1 and Ki-67. In the consecutive clinical specimens of ESCC, accompanying by the increased expression status of PLCE1, Ki-67, and CD34 staining, the intensity score of their staining was increased (Fig. 2e). Moreover, the MVD numbers and the score of Ki-67 expression were much higher in PLCE1(+) ESCC tissues than in PLCE1(−) ESCC tissues (Fig. 2f). Altogether, these results suggest that PLCE1 may paticipate in angiogenesis and promote proliferation in ESCC. Survival analysis showed that high MVD in ESCC patients meant significantly shorter survival compared to low MVD (Fig. 2g). Survival was reduced when Ki-67 was positive compared to those with a negative expression of Ki-67 (Fig. 2h). Thus, PLCE1 may contribute to an oncogenic role in angiogenesis and proliferation of ESCC.

### PLCE1 has strong proto-oncogene function and promotes angiogenesis in ESCC in vitro and in vivo

**Fig. 3:**
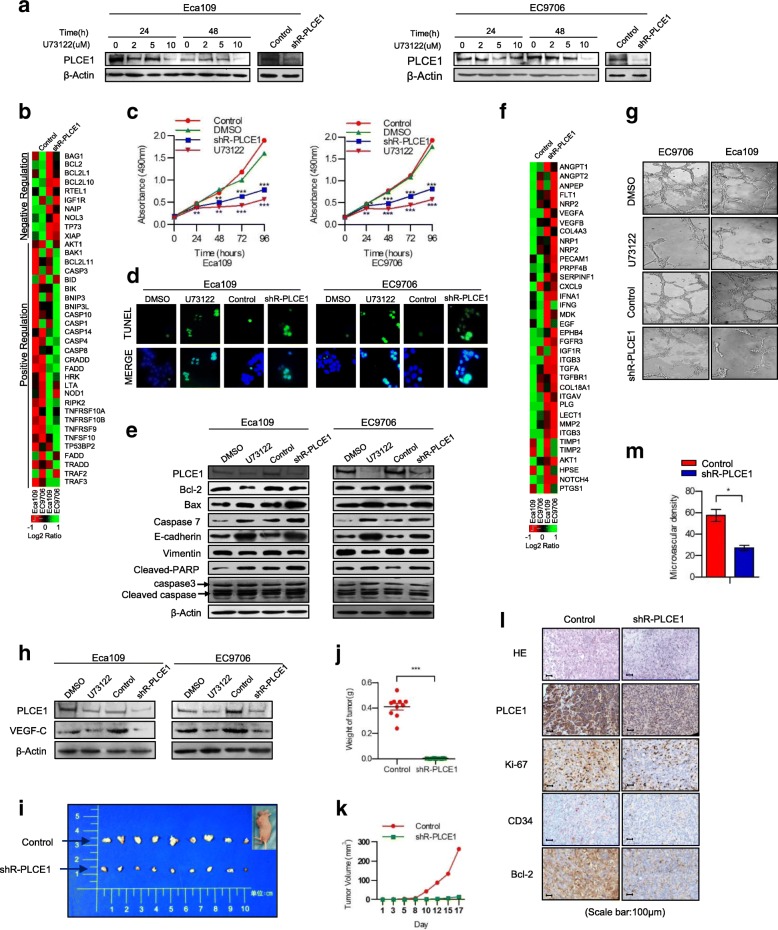
PLCE1 promotes proliferation and angiogenesis of ESCC cells in vitro and induces aggressiveness in vivo. **a** Eca109 and EC9706 cells treated with U73122 at 0, 2, 5, and 10 concentrations for 24 and 48 h. ShR-PLCE1 were transfected at a MOI of 15 for 60 h. PLCE1 expression measured by Western blot. **b** Real-time PCR analysis demonstrating relationship between PLCE1 expression and apoptosis. Color represents intensity scale for vector of PLCE1 shRNA versus control, as calculated by log2 transformation. **c** Eca109 and EC9706 cells treated shR-PLCE1 or U73122 at the indicated concentration for 0, 24, 48, 72, and 96 h. Cell viability measured by MTT and presented as means ± SD from three separate experiments. **d** TUNEL staining of cells treated as indicated; **e** shR-PLCE1 on apoptosis-related proteins assayed by Western blot. β-Actin was a loading control. **f** Real-time PCR analysis demonstrated the positive relationship between PLCE1 expression and angiogenesis. Pseudo-color represents intensity scale for the vector or PLCE1 shRNA versus control, as calculated by log2 transformation. **g** Tube formation by indicated cells. **h** Effects of shR-PLCE1 on VEGF-C protein expression as detected by Western blot. **i** Xenograft model in nude mice; representative images of tumors from all mice in each group. Mean tumor weights **j** and tumor volume growth curves **k** for tumors formed by the indicated cells. **l** H&E and IHC staining demonstrated that PLCE1 induced the aggressive phenotype of ESCC cells in vivo. Scale bar, 100 μm. Microvascular density **m** show that PLCE1 promotes resistance to apoptosis and angiogenesis in vivo. All data are presented as mean ± SD. *P < 0.05, ***P < 0.001

Inhibited endogenous PLCE1 expression significantly reduced cell proliferation compared with the controls and significantly increased the apoptosis-related moleculars expression (Fig. 3a-c). Colony formation assays revealed that the transfection of Eca109 and EC9706 cells with shR-PLCE1 or U73122 reduced growth compared with controls or blanks (Additional file 2: Figure S1a, b). Annexin V (AV)– FITC and TUNEL assays demonstrated that silencing PLCE1 promoted apoptosis in ESCC cells (Fig. 3d, Additional file 2: Figure S1c-e). In Eca109 and EC9706 cells, after treatment with shR-PLCE1 or U73122, red fluorescence emitted by JC-1 was reduced, but green fluorescence was elevated, indicating PLCE1 induced mitochondrial dysfunction in ESCC cells (Additional file 2: Figure S1f). Western blot confirmed that the expression of bax, caspase 3, caspase 7, and cleaved poly (ADP-ribose) polymerase (PARP), which positively regulate apoptosis, were upregulated in the PLCE1-silenced cells. Bcl-2 was downregulated and E-cadherin was upregulated in the PLCE1-silenced cells, but vimentin was downregulated (Fig. 3e). Thus, PLCE1 is key to tumorigenicity of ESCC cells in vitro. Real-time PCR Chip analysis demonstrated a positive relationship between PLCE1 expression and angiogenesis-related molecules (Fig. 3f). We also observed that silencing PLCE1 strongly inhibits Eca109 and EC9706 ESCC cell induction of human umbilical vein endothelial cells (HUVECs) proliferation (Additional file 3: Figure S2a-c). Inhibition of PLCE1 significantly reduced the ability of ESCC cells to induce tubule formation and migration by HUVECs in vitro (Fig. 3g, Additional file 3: Figure S2d). Multiple studies have reported that expression level of VEGF-C, a vital pro-angiogenic factor, correlates strongly with ESCC progression and acts as an independent prognostic factor for ESCC. Additional file 3: Figure S2e shows *VEGF-C* mRNA expression was downregulated in PLCE1-silenced cells, and Western blot data agreed with this (Fig. 3h). Thus, PLCE1 contributes to promoting angiogenesis in ESCC cells.

Moreover, the tumors formed by PLCE1-silenced cells were smaller compared with tumors formed by controls in Fig. 3i–k. IHC staining indicated that PLCE1-silenced tumors had low Ki-67, CD34, and Bcl-2 expression and decreased MVD (Fig. 3l, m). IF staining showed that PLCE1 knockdown increased TUNEL-positive apoptotic cells compared with the controls (Additional file 3: Figure S2f, g). Thus, PLCE1 contributes to progression of ESCC in vivo.

### PLCE1 regulates the NF-κB through PI-PLCε signaling pathway in ESCC

**Fig. 4:**
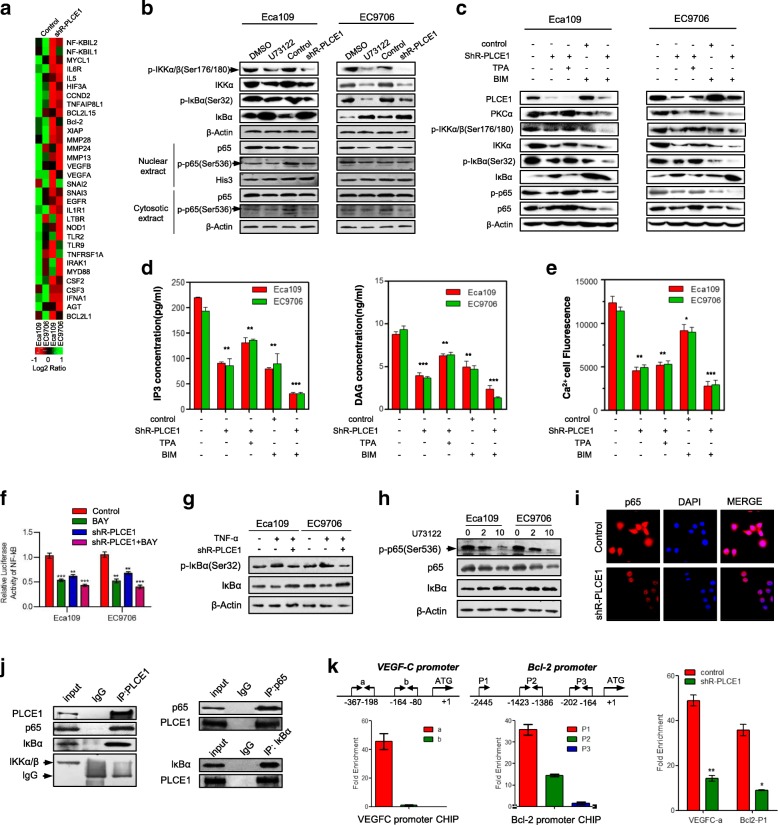
PLCE1 activates the NF-κB signaling pathway in ESCC. **a** Real-time PCR analysis shows overlap between NF-κB-dependent gene expression and PLCE1-regulated gene expression. Color represents intensity for vector of PLCE1 shRNA versus control, as calculated by log2 transformation. **b** Western blot for indicated proteins in indicated cells. **c** Western blot analysis was performed to indicate PLCE1 affect NF-κB signaling pathway through PI-PLCε pathway by using PLCE1 shRNA, TPA, and BIM. **d** ELISA assays showed the effects of PLCE1 shRNA, TPA, and BIM on intracellular levels of the PI-PLCε-pathway-related proteins IP3 and DAG. **e** Laser scanning confocal microscopy was used to measure intracellular calcium fluorescence pixel values of ESCC cells after treatment with PLCE1 shRNA, TPA, and BIM, which triggered positive and negative effects on the PI-PLC pathway. The histogram shows semi-quantitative analysis of fluorescence. **f** Activity of NF-κB luciferase reporter gene in ESCC cells expressed with indicated treatment. **g** IκBα and p-IκBα (Ser 32) in indicated cells. **h** Effects of 48 h U73122 treatment at 0, 2, and 10 concentrations on protein levels of phosphorylated p65 (Ser536); p65 and IκBα in ESCC cells. **i** IF analysis of Eca109 and EC9706 cells transfected with shR-PLCE1. Cells were fixed, stained with antibodies to p65 (red) and by 4′,6-diamidino-2-phenylindole (blue), incubated with appropriate secondary antibodies, and analyzed using double IF assays. **j** Whole cell lysates from Eca109 and EC9706 cells immunoprecipitated with antibodies against indicated proteins. **k** ChIP assay p65-binding sites on Bcl2 and VEGF-C genes. Extent of recruitment assessed by real-time PCR

As growing body of evidence demonstrates that the NF-κB signaling pathway plays a central role in both angiogenesis and resistance to apoptosis in cancer, we investigated whether PLCE1 participates in regulation of the NF-κB signaling pathway in ESCC. As shown in Fig. 4a, Real-time PCR Chip analysis shows that inhibition of PLCE1 significantly reduced NF-κB downstream genes, which suggest that PLCE1 may contribute to activation of NF-κB signaling pathway-related molecules. We observed that the downregulation of PLCE1 decreased, subsequently decreasing phosphorylated-IKK-α/β and-IκBα protein. The level of Nuclear NF-κB p65 protein decreased in PLCE1-silenced cells (Fig. 4b), suggesting that PLCE1 regulates NF-κB transcriptional activity by promoting NF-κB “cytoplasmic-nuclear” translocation. To clarify the specific molecular mechanism on how PLCE1 affects NF-κB, we tested whether PI-PLCε signaling pathway, which hydrolyzes plasma membrane phosphatidylinositol 4,5-bisphosphate (PIP2) to produce inositol trisphosphate (IP3) and diacylglycerol (DAG), was involved. When the ESCC cells were treated with 12-O-tetradecanoylphorbol 13-acetate (TPA), a regular substitution of diacylglycerol (DAG), results showed it could rescue the side effect of PLCE1 knockdown in NF-κB-related proteins, phosphorylated-IKK-α/β, −p65, and-IκBα protein. When cells were treated with bisindolylmaleimide (BIM), a broad-specificity DAG-dependent activation of protein kinase Cα (PKCα) inhibitor, it showed a similar effect to PLCE1 knockdown (Fig. 4c). As the great cleaved products after PLCE1 activated, IP3 and DAG were measured by ELISA assays. The level of IP3 and DAG in ESCC cells showed a significant decrease after treated with shR-PLCE1 (Fig. 4d). IP3-dependent calcium release was also observed through laser confocal microscopy. PLCE1 knockdown also triggers the downregulation of Ca^2+^ content as well (Fig. 4e, Additional file 4: Figure S3a). Silencing of PLCE1 after bay-11-7082 treatment (individually or in combination) reduced NF-κB luciferase reporter gene activity (Fig. 4f). IκBα-S32 phosphorylation antibody showed increased phosphorylation after TNFα treatment. However, silencing PLCE1 expression significantly decreased expression of IκBα-S32 phosphorylation (Fig. 4g). Consistently, typical NF-κB signaling gene protein and the phosphorylation of p65 in ESCC cells were downregulated by U73122 (Fig. 4h). Endogenous p65 was localized to the nucleus of controls, whereas p65 nuclear localization significantly decreased in shR-PLCE1-infected Eca109 cells (Fig. 4i). Immunoprecipitation (IP) assay shows that anti-PLCE1 bound to a band in p65 and IκBα IPs, which migrated together with PLCE1 protein in cell lysates (Fig. 4j left). Conversely, p65 and IκBα antibodies bound to PLCE1 protein in anti-p65 and anti-IκBα antibody (Fig. 4j right). Thus, PLCE1 and p65 and IκBα proteins interact. Chromatin IP (ChIP) experiments shows that p65 binding to the VEGF-C promoter region (− 367/− 198) increased after PLCE1 exposure.These results suggest that PLCE1 induces the recruitment of p65 to the promoter region of endogenous VEGF-C gene, and leads to VEGF-C expression (Fig. 4k left). Similarly, we also observed that PLCE1 induces p65 to the promoter region (− 2445) of the endogenous bcl-2 gene and leads to bcl-2 expression (Fig. 4k right).

### PLCE1 enhances proliferation and angiogenesis via activation of the NF-κB signaling pathway in ESCC in vivo and in vivo

**Fig. 5:**
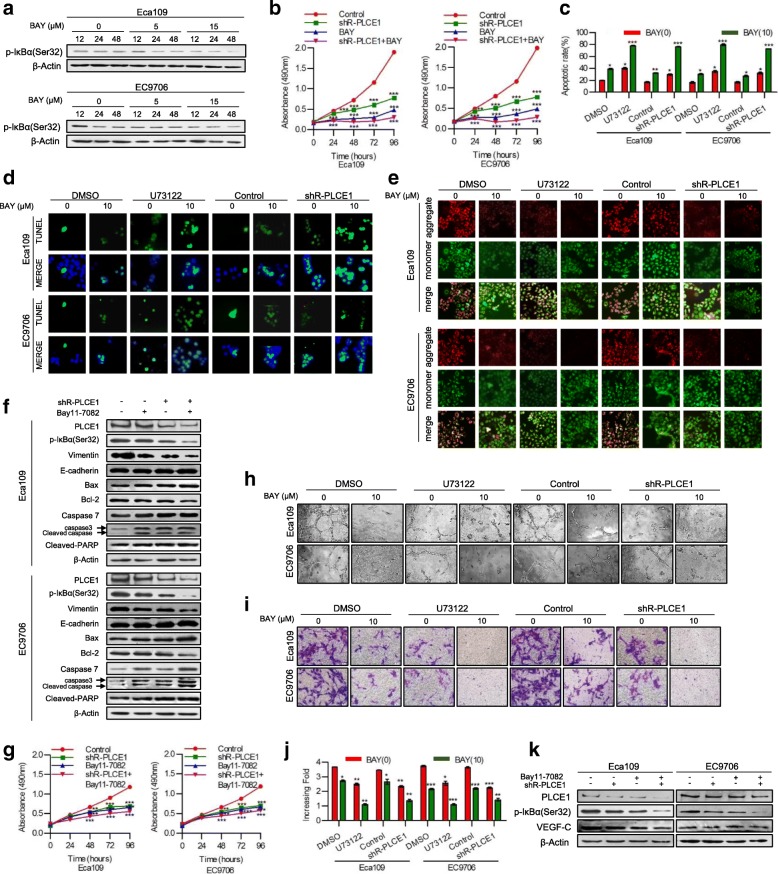
PLCE1 inhibits apoptosis and enhances angiogenesis via activation of the NF-κB signaling pathway in ESCC in vitro. **a** Eca109 and EC9706 cells treated with Bay11–7082 at the 0, 5, 15 concentrations for 12, 24 and 48 h. p-IκBα(Ser32) expression measured by Western blot. **b** Eca109 and EC9706 cells were treated with shR-PLCE1 or/and Bay11–7082 at the indicated concentration for 0, 24, 48, 72, and 96 h. Cell viability was measured by MTT and presented as means ± SD from three separate experiments. **c** FITC–PI staining of cells treated with shR-PLCE1 or U73122 or/and Bay11–7082 and results means ± SD from three independent experiments. **d** TUNEL staining of cells treated with indicated concentration. **e** Eca109 and EC9706 cells were treated with shR-PLCE1 or U73122 or/and Bay11–7082 and then subjected to JC-1 staining assays. **f** Effects of shR-PLCE1 and Bay11–7082 on apoptosis-related proteins assayed by Western blot. β-Actin was loading control. **g** MTT assay after stimulation as indicated. **h** Tube formation in cells. **i** Representative images and **j** quantification of cell invasion by indicated cells in transwell matrix penetration assay. Bar represents mean ± SD of three independent experiments. *P < 0.05, **P < 0.01, ***P < 0.001. **k** VEGF-C protein expression with indicated treatments, and cells using Western blot

Therefore, we hypothesized that PLCE1 regulates apoptosis and angiogenesis via the NF-κB signaling pathway in ESCC cells. To test this hypothesis, we first used NF-κB inhibitor Bay11–7082 to inhibit the NF-κB signaling pathway. Data show that bay11–7082 exerted time- and dose-dependent inhibition of NF-κB signaling pathway in human ESCC (Fig. 5a). PLCE1 knockdown in infected Eca109 and EC9706 ESCC cells increased the effects of NF-κB inhibitor, reducing proliferation and increased apoptosis (*P* < 0.05, Fig. 5b-f, Additional file 5: Figure S4a, b). PLCE1 knockdown in infected Eca109 and EC9706 ESCC cells increased the effects of NF-κB inhibitor, reducing HUVEC proliferation and ESCC cells’ ability to induce tubule formation and migration by HUVECs in vitro (*P* < 0.05, Fig. 5g-k, Additional file 5: Figure S4c).

**Fig. 6:**
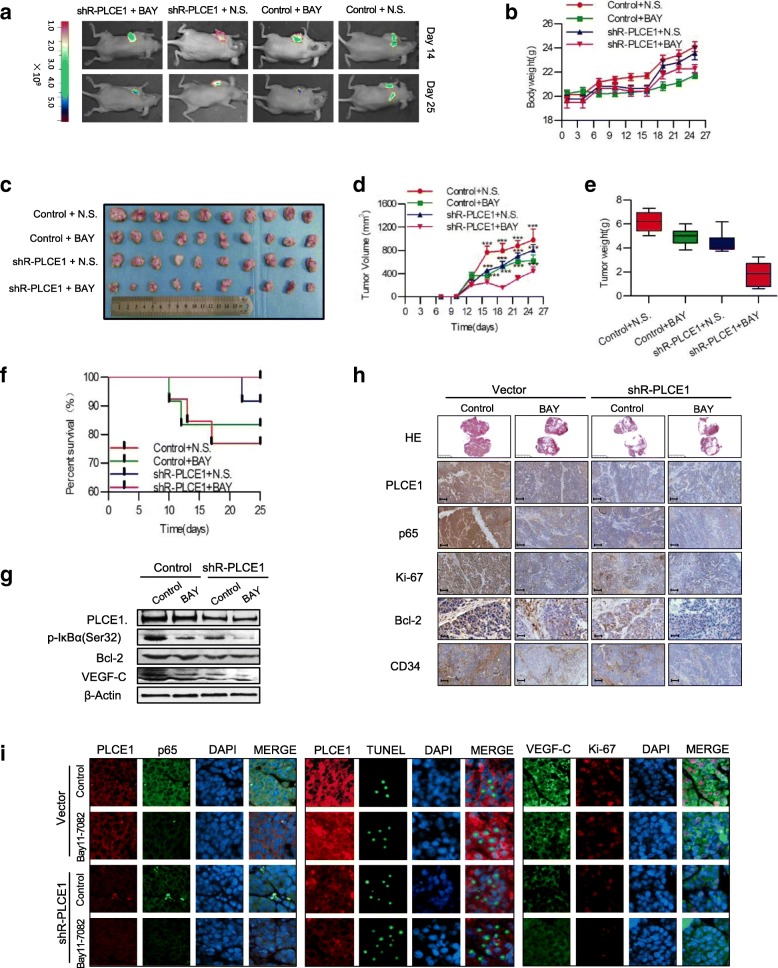
PLCE1 enhances angiogenesis and inhibits apoptosis via activation of the NF-κB signaling pathway in ESCC in vivo. **a** Xenograft model in nude mice; tumor images from all mice on days 14 and 25. Body weight **b**, tumor volume growth curves **c**, **d** and mean tumor weights **e** for tumors formed by indicated cells. **f** Survival, **g** Expression of p-IκBα (Ser32), Bcl-2, and VEGF-C in nude mouse tissue. **h, i** H&E and IHC staining and IF confirm PLCE1 induced aggressive phenotype of ESCC cells in vivo, and Bay11–7082 can reverse this. HE: Scale bar, 5 mm; PLCE1, p65, Ki-67, CD34, Scale bar, 100 μm; Bcl-2, Scale bar, 50 μm

We next sought to determine whether NF-κB inhibition affected tumor growth in PLCE1-silenced Eca109 cells subcutaneous tumor model. Figure 6a shows bioluminescent images of xenografts at different time points and treatment. On day 25, we observed metastatic signals in controls and shR-PLCE1-Bay11–7082-treated groups and tumor number and size decreased significantly (Fig. 6a). Tumor-bearing mice treated with Bay11–7082 had reduced tumor volume and weight compared with controls (*P* < 0.05, Fig. 6b-e). During the experiment, few nude mice appearance cachexia status, and before the end of the experiment, treatment and control groups emerge different number of deaths and the shR-PLCE1-Bay11–7082-treated group has no die (Fig. 6f). We confirmed the decreased expression of VEGF-C and Bcl-2 by Western blot (Fig. 6g) and immunohistochemistry for PLCE1, p65, Ki-67, Bcl-2, and CD34 showed reduced expression of all three proteins in Bay11–7082-treated xenografts (Fig. 6h, i). These results confirm our in vitro observations.

### Clinical relevance of PLCE1-induced NF-κB activation in ESCC

**Fig. 7:**
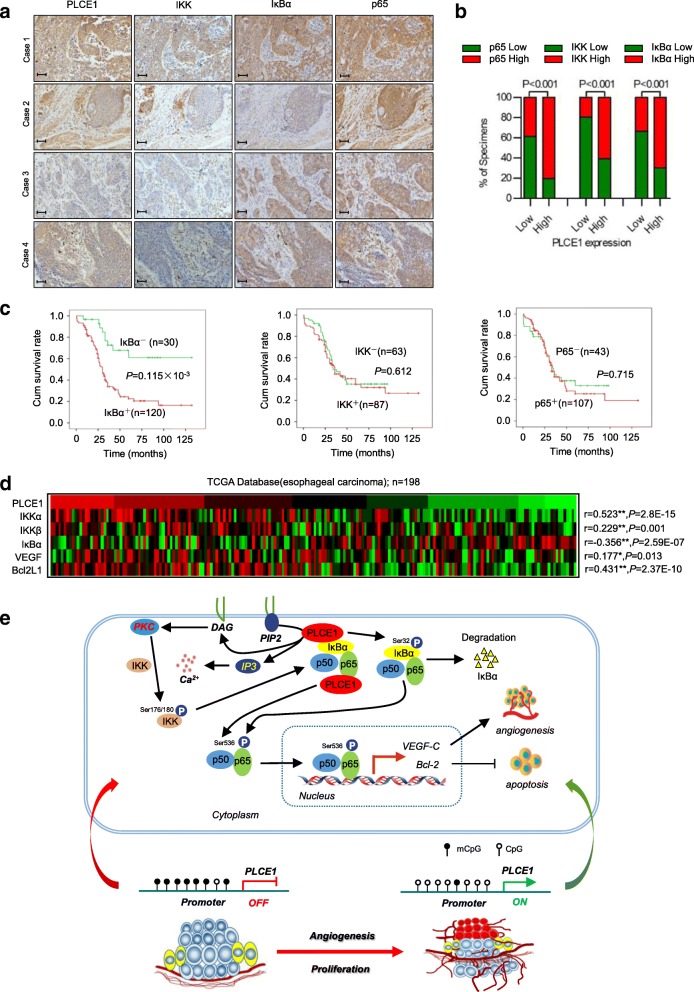
Clinical relevance of PLCE1-induced NF-κB activation in ESCC. **a**, **b** PLCE1 were associated with NF-κB molecules expression in 368 primary human ESCC specimens. Scale bar, 100 μm. **c** Kaplan-Meier curves of patients with ESCCs with low versus high expression of IκBα, IKK and p65; (*P* = 0.115 × 10^− 3^, *P* = 0.612 and *P* = 0.715, respectively, log-rank test) **d** *PLCE1* mRNA expression was correlated positively with IKKα, IKKβ, Bcl2L1, and *VEGF* mRNA expression and negatively with IκBα mRNA expression in published profiles of ESCC (*n* = 198; *P* < 0.05; TCGA database of esophageal carcinoma). **e** Proposed model. Schematic model of the regulatory pathway involving PI-PLCε-NF-κB signaling pathway in ESCC. PLCE1 activates the PI-PLCε-NF-κB signaling pathway, enhances angiogenesis, and inhibits apoptosis, which consequently leads to progression of ESCC

Finally, we examined whether PLCE1/NF-κB axis was also clinically relevant. ESCC tissue specimens showed that PLCE1 expression was positively correlated with p65, IKK, and IκBα expression (*P* < 0.001, *P* < 0.001, and *P* < 0.001, respectively; Fig. 7a, b). Consistently, patients with higher IκBα expression had shorter survival (*P* = 0.115 × 10^− 3^; Fig. 7c). Figure 7d shows PLCE1 expression in The Cancer Genome Atlas (TCGA) database of 198 collected clinical esophageal carcinoma samples correlated positively with IKKα expression (*r* = 0.523, *P* = 2.8E-15) and IKKβ expression (*r* = 0.229, *P* = 0.001) and negatively with IκBα expression (*r* = − 0.177, *P* = 0.013). These data suggest that epigenetically upregulated oncoprotein PLCE1 can activate the NF-κB signaling pathway and may promote angiogenesis and cause poor clinical outcomes in ESCC (Fig. 7e).

## Discussion

PLCE1 is a multifunctional signaling protein that may act as an oncoprotein, promoting malignant transformation of primary cell lines, tumor growth, migration, and metastasis in various human cancers [7, 8]. Previous work confirmed a greater expression of PLCE1 protein in homozygous mutant types of rs12263737 and rs2274223 carriers than in homozygous wild-type control carriers [6, 26]. DNA hypomethylation is a key switch that controls gene expression. We previously indicated that miR-34a [27] and miR-203 inactivation are correlated with CpG hypermethylation in Kazakh patients with ESCC. We also noted that elevated PLCE1 expression in ESCC tissues was due to promoter hypomethylation and CpG_5.6 hypomethylation was correlated with unfavorable prognosis. Thus, epigenetically upregulated PLCE1 is essential for its transcription, which can cause epigenetic activation, enzyme activity, and augmentation of inflammation esophageal epithelia. Zhai’s group showed that CRISPR/Cas9-mediated mutations of PLCE1 decreased transcriptional activity of snails, thereby inhibiting cell migration and invasion in vitro and in vivo [11]. The addition of anti-PLCE1 antibody increased the expression of p53 in NSCLC cells, increasing apoptotic NSCLC cells [28]. Li’s work showed that PLCE1 significantly decreased apoptosis by modulating p53 promoter methylation in esophageal cancer cells [29]. Our results suggested that PLCE1 can activate the NF-κB signaling pathway, promote p65-mediated transcription, and recruit Bcl-2 and VEGF-C promoters, thereby inhibiting apoptosis and enhancing angiogenesis. Overall, the induction of PLCE1 degradation by hypermethylation may be a therapeutic strategy for preventing PLCE1 activity and treating esophageal cancer.

Inflammation is necessary for tumor occurrence and development. Different PLC families share catalytic properties and are characterized by distinct regulatory interactions. These families may be related to the inflammatory tumor microenvironment. PLCγ2 is highly expressed in immune cells and regulates their activation, inducing immune inflammatory reactions. PLCδ1 negatively regulates expression of pro-inflammatory cytokines, such as interleukin (IL)-1b in keratinocytes [30, 31]. Ikuta’s group reported that PLCE1-deficient mice have resistance to 12-O-tetradecanoylphorbol-13-acetate (TPA)-induced skin inflammation [32]. Chronic inflammation is thought to rise to malignancy [33]. Clinical and epidemiological studies suggest chronic inflammation as a major risk factor for cancers, including ESCC [33, 34]. Chronic inflammation can trigger the development of esophageal squamous dysplasia and eventually ESCC [35]. As a classical inflammatory pathway, NF-κB plays important roles in the progression of various human cancers, and IκBs were degraded after phosphorylation and ubiquitination, whereas p50/p65 nuclearly translocated, activating transcription of various downstream genes [16–19, 36, 37]. But the link between PLCE1 and inflammation-related cancer is not well understood. Our studies showed that NF-κB activation plays vital roles in the development and progression of ESCC, and NF-κB pathway blockade can inhibit proliferation and suppress angiogenesis and metastasis in ESCC. However, how genes act on the NF-κB signaling pathway is not clear. Zhu’s study suggested that TAR DNA-binding protein 43 can compete with p65 in binding with KPNA4 nuclear transporter to reduce translocation, which allows the nuclear presence of p65 [38]. Golgi phosphoprotein 3 may promote ubiquitin conjugation of NF-κB signaling-related proteins and sustain NF-κB activation [39]. Our results showed that PLCE1 can phosphorylate IKK. The direct binding of IκBα and p65 promotes IκBα-S32 and p65-S536 phosphorylation, which promotes the translocation of p65 to the nucleus. p65, as a transcription factor, can directly bind VEGF-C and bcl-2 promoters, enhancing angiogenesis and inhibiting apoptosis. Similar results were observed by Guo’s group who showed that TGR5 activation can suppress IκBα phosphorylation and nuclear translocation of p65 [40].

We report that PLCE1 regulates NF-κB-mediated transcription in ESCC and that PLCE1 enhances angiogenesis and inhibits apoptosis by activating the PI-PLCε-NF-κB pathway. PI-PLC signaling pathway showed a broad effect on cell biology. Once activated, PLCE1 cleaves PIP2 into DAG and IP3, which further triggers IP3-dependent calcium release in the nucleus and PKC, which involved in cellular physiological function as two critical regulators [41, 42]. Our data suggest, after knockdown of PLCE1, the levels of PI-PLCε-signaling-pathway-related proteins, DAG, IP3, and PKCα, were downregulated, which suggests PLCE1 plays an important role in PIP2 cleavage. DAG and IP3, the cleaved products of PIP2, have abroad effects on cell behavior. DAG activates PKC pathways, which has been shown to activate NF-κB pathway and further affect the proliferation of cancer cells [43]. IP3, on the other hand, mobilizes internal Ca^2+^ stores. In this study, we measured the content of cellular Ca^2+^ through laser confocal microscopy. The level of Ca^2+^ also downregulated in ESCC cells. Recently, studies showed the release of Ca^2+^ stores to be related to cell adhesion [44]. After treatment with thapsagargin in epithelial cells, Ca^2+^ stores releases enhance the formation of lamellipodia [45]. Furthermore, Marc D. Basson et al. found that the induced Ca^2+^ flux activate PKCβ and NF-κB to promote proliferation in breast and prostate cancer cells [46]. Together, we preliminarily clarified PLCE1 works as a phosphatase through PI-PLCε and so activates the NF-κB signaling pathway. Our previous work indicated that PLCE1 expression was positively correlated with NF-κB-related proteins in Kazakh patients with ESCC [22]. Du’s group reported that PLCE1 promotes renal cell carcinoma cell growth via the NF-κB-mediated upregulation of VEGF [23]. A similar mechanism has been proven in colon epithelial cells, whereby PLCE1 can activate the NF-κB pathway via PKD-PEA15-RSK to facilitate inflammation and inflammation-associated carcinogenesis [24]. Guo revealed that compared with the wild-type mouse esophagus, the mRNA of cytokines (e.g., TNFα, NF-κB, IL-1β, IFN-γ, and IL-6) was significantly decreased in PLCE1^−/−^ mouse esophagus [47]. Abnet reported that PLCE1 can promote intestinal tumorigenesis by inducing inflammation and angiogenesis in a transgenic mouse model [5]. Thus, our preliminary results indicated the possible regulatory mechanism by which PLCE1 promotes NF-κB expression and induces p65 transcriptional regulation capability on Bcl-2 and VEGF-C via interaction at binding sites.

Vasculature is recognized as another vital constituent of local environment sustaining tumor development. Without the necessary microenvironment for neovascularization, tumor growth is arrested [48]. Micro-vascular pattern and changes in the morphology of intrapapillary capillary loops on magnification endoscopy is a reliable indicator of tissue atypia and provides evidence for early-stage squamous cell carcinoma of the esophagus [49, 50]. Solid tumor angiogenesis is complex and an angiogenic switch is required to sustain tumorigenesis [51]. The VEGF family has been identified as specific angiogenic and lymphangiogenic factors in tumor proliferation, angiogenesis, invasiveness, and metastasis [52]. VEGF-A, as an angiogenic switch, occurs at the early stage of tumorigenesis around low-grade adenoma formation in PLCE1^−/−^ mice [13]. Huang et al. recently discovered that IL-6-exposed SV-LEC Src-mediated ERK1/2 and p38 mitogen-activated protein kinase activation resulted in the binding of p65 to the promoter region of VEGF-C, leading to VEGF-C expression [53]. We report a mechanism in ESCC, whereby after the activation of the NF-κB signaling pathway, p65 transfers to the nucleus and binds to the VEGF-C promoter region (− 367/− 198) as a transcription factor, and binding ability increased after PLCE1 exposure. As an angiogenic switch, PLCE1-mediated activation of NF-κB /VEGF-C signaling is involved in the angiogenesis of esophageal cancer, which not only supplies nutrients and oxygen to proliferative tumor cells, but also serves as the conduit for migration.

In conclusion, the hypomethylation-associated upregulation of PLCE1 expression is closely correlated with tumor angiogenesis and poor prognosis in ESCC cohorts. PLCE1 can activate NF-κB through PI-PLCε signaling pathway. Furthermore, PLCE1 can directly bind p65 and IκBα proteins, thus promoting IκBα-S32 and p65-S536 phosphorylation. Consequently, phosphorylated IκBα promotes translocation of p50/p65 to the nucleus and the p65, as a transcription factor, can directly bind vascular endothelial growth factor-C and bcl-2 promoters, thereby enhancing angiogenesis and inhibiting apoptosis in vitro. Moreover, we demonstrated that the downregulation of PLCE1 can induce apoptosis and inhibit angiogenesis of ESCC cells in vitro and in vivo by inhibiting the NF-κB signaling pathway. Overall, our data reveal a novel mechanism for epigenetically upregulated PLCE1 in driving angiogenesis and proliferation in ESCC by activating the NF-κB signaling pathway, and suggest that modulation of PLCE1 may provide a therapeutic approach for combating ESCC.

## Additional files

Additional file 1: **Table S1.** Correlation between DNA methylation and PLCE1 expression in ESCC tissues; **Table S2.** Correlation between the expression and promoter methylation of PLCE1 in ESCC tissues; **Table S3.** Compare the CpG sites methylation of PLCE1 between ESCC and NCAT tissues; **Table S4.** Sequences of PCR primers used in this study. (DOCX 26 kb)

Additional file 2: **Figure S1.** PLCE1 promotes proliferation of esophageal carcinoma cell lines. **a** Colony formation of ESCC cells decreased by shR-PLCE1 or U73122 treatment and **b** are means ± SD from three separate experiments. **c** AV-FITC–PI staining of cells treated as indicated **d** means ± SD from three independent experiments. **e** quantification of TUNEL-positive cells. *P < 0.05, **P < 0.01, ***P < 0.001. **f** Eca109 and EC9706 cells were treated as indicated and JC-1 staining assay. (PDF 363 kb)

Additional file 3: **Figure S2.** PLCE1 promotes angiogenesis in ESCC cells in vitro and induces aggressiveness in vivo. **a, b** MTT assay after stimulation with conditioned medium from indicated cells. **c** Quantification of generated tubes. **d** Representative images (left panel) and quantification (right panel) of cell invasion by indicated cells in transwell matrix penetration assay. Each bar represents mean ± SD of three independent experiments. **P < 0.01, ***P < 0.001. **e** Effects of shR-PLCE1 on VEGF-C mRNA expression as detected by real-time PCR analysis. **f, g** IF show that PLCE1 promotes resistance to apoptosis and angiogenesis in vivo. All data are presented as mean ± SD. *P < 0.05, ***P < 0.001. (PDF 273 kb)

Additional file 4: **Figure S3.** PLCE1 ablation reduces the IP3-dependent intracellular calcium release. **a** Laser scanning confocal microscopy was used to measure intracellular calcium fluorescence pixel values of ESCC cells after treatment with PLCE1 shRNA, TPA, and BIM. (PDF 95 kb)

Additional file 5; **Figure S4.** PLCE1 inhibits apoptosis and enhances angiogenesis via activation of the NF-κB signaling pathway in ESCC in vitro. **a** FITC–PI staining of cells treated with shR-PLCE1 or U73122 or/and Bay11–7082 and results means ± SD from three independent experiments. **b** quantification of TUNEL-positive cells. *P < 0.05, **P < 0.01, ***P < 0.001. **c** Tube formation in cells quantification of generated tubes. (PDF 156 kb)

## Abbreviations

bcl-2: B-cell lymphoma-2
BIM: Bisindolylmaleimide
CRISPR/Cas9: Clustered regularly interspaced short palindromic repeats/Cas9
DAG: Diacylglycerol
ESCC: Esophageal squamous cell carcinoma
FLIP: FLICE inhibitory protein
GWAS: Genome-wide association studies
IKK: IkB kinase
IP3: Inositol trisphosphate
MALDI-TOF MS: Matrix-Assisted Laser Desorption/ Ionization Time of Flight Mass Spectrometry
MMP: Mitochondrial membrane potential
MTT: 3-(4,5-Dimethylthiazol-2-yl)-2,5-diphenyltetrazolium bromide
MVD: Microvessel density
NF-κB: Nuclear factor-κB
PIP2: Phosphatidylinositol 4,5-bisphosphate
PI-PLC: Phosphoinositide-phospholipase C
PKC: Protein kinase C
PLCE1: Phospholipase C epsilon 1
SNPs: Single-nucleotide polymorphisms
VEGF-C: Vascular Endothelial Growth Factor C
