# Supplementary material for: Epigenetically upregulated oncoprotein PLCE1 drives esophageal carcinoma angiogenesis and proliferation via activating the PI-PLCε-NF-κB signaling pathway and VEGF-C/ Bcl-2 expression
Source: Mol Cancer. 2019 Jan 4;18:1. doi: 10.1186/s12943-018-0930-x (PMC6320601; doi:10.1186/s12943-018-0930-x)
Supplement: Supplementary file 1 — Table S1. Correlation between DNA methylation and PLCE1 expression in ESCC tissues; Table S2. Correlation between the expression and promoter methylation of PLCE1 in ESCC tissues; Table S3. Compare the CpG sites methylation of PLCE1 between ESCC and NCAT tissues; Table S4. Sequences of PCR primers used in this study. (DOCX 26 kb) [file 12943_2018_930_MOESM1_ESM.docx]

| **Table S1.** Sequences of PCR primers used in this study. | | |
| --- | --- | --- |
| Gene | Primer | Taregt Length |
| PLCE1 | For:5’-aggaagagagGTTGGGTATATTGATGGGGTTTAAT-3’  Rev:5’-cagtaatacgactcactatagggagaaggctACCCCTAAAAACCATCCTTTCTAAC-3’ | 320bp |

“For”: Forward, “Rev”: Reverse.

| **Table S2** Compare the CpG sites methylation of PLCE1 between ESCC and and NCAT tissues | | | | | | |
| --- | --- | --- | --- | --- | --- | --- |
| CpG Site | ESCC | | Normal | | Z | *P* |
|  | N | 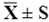 | N | 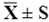 |  |  |
| CpG_2 | 59 | 0.2025±0.1518 | 48 | 0.3238±0.1245 | -4.498 | **0.000***** |
| CpG_3 | 131 | 0.0950±0.0708 | 104 | 0.0897±0.0582 | -0.017 | 0.986 |
| CpG_4 | 132 | 0.0651±0.0702 | 104 | 0.0599±0.0541 | -0.336 | 0.737 |
| CpG_5.6 | 129 | 0.1184±0.0594 | 104 | 0.1389±0.0839 | -2.073 | **0.038*** |
| CpG_7.8 | 123 | 0.0807±0.0493 | 101 | 0.1052±0.0604 | -3.588 | **0.000***** |
| CpG_9.10 | 131 | 0.0720±0.0512 | 104 | 0.0937±0.0584 | -2.931 | **0.003**** |

N stand for the number of analyzed CpG units; * represents *P*<0.05，** indicates *P*<0.01，*** depicts *P*<0.001.

| **Table S3** Correlation between DNA methylation andPLCE1 expression in ESCC tissues | | | | | |
| --- | --- | --- | --- | --- | --- |
| CpG Site | N | PLCE1 methylation  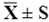 | PLCE1 expression  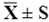 | *r* | *P* |
| CpG_2 | 59 | 0.2025±0.1518 | 6.0339±3.0341 | -0.267 | **0.041*** |
| CpG_5.6 | 129 | 0.1184±0.0594 | 6.1938±2.9582 | -0.216 | **0.003**** |
| CpG_7.8 | 123 | 0.0807±0.0493 | 6.2114±2.9456 | -0.118 | 0.193 |
| CpG_9.10 | 131 | 0.0720±0.0512 | 6.1603±2.9478 | -0.140 | 0.112 |

Bivariate correlation analysis (two-sided), N stand for the number of analyzed CpG units; * represents *P*< 0.05, ** indicates *P*< 0.01.

| **Table S4** Correlation between the expression and promoter methylation of PLCE1 in ESCC tissues | | | | | |
| --- | --- | --- | --- | --- | --- |
| PLCE1 Expression | N | Amplicon Methylation | | *χ^2^* | *P* |
|  |  | Hypomethylated | Methylated |  |  |
| High | 70 | 49(70.0%) | 21(30.0%) | 3.932 | **0.047*** |
| Low | 62 | 33(53.2%) | 29(46.8%) |  |  |

N stand for the number of analyzed patients; ** represents *P*<0.001.
