# Supplementary figures and images for: Epigenetically upregulated oncoprotein PLCE1 drives esophageal carcinoma angiogenesis and proliferation via activating the PI-PLCε-NF-κB signaling pathway and VEGF-C/ Bcl-2 expression
Source: Mol Cancer. 2019 Jan 4;18:1. doi: 10.1186/s12943-018-0930-x (PMC6320601; doi:10.1186/s12943-018-0930-x)

Fig. S1

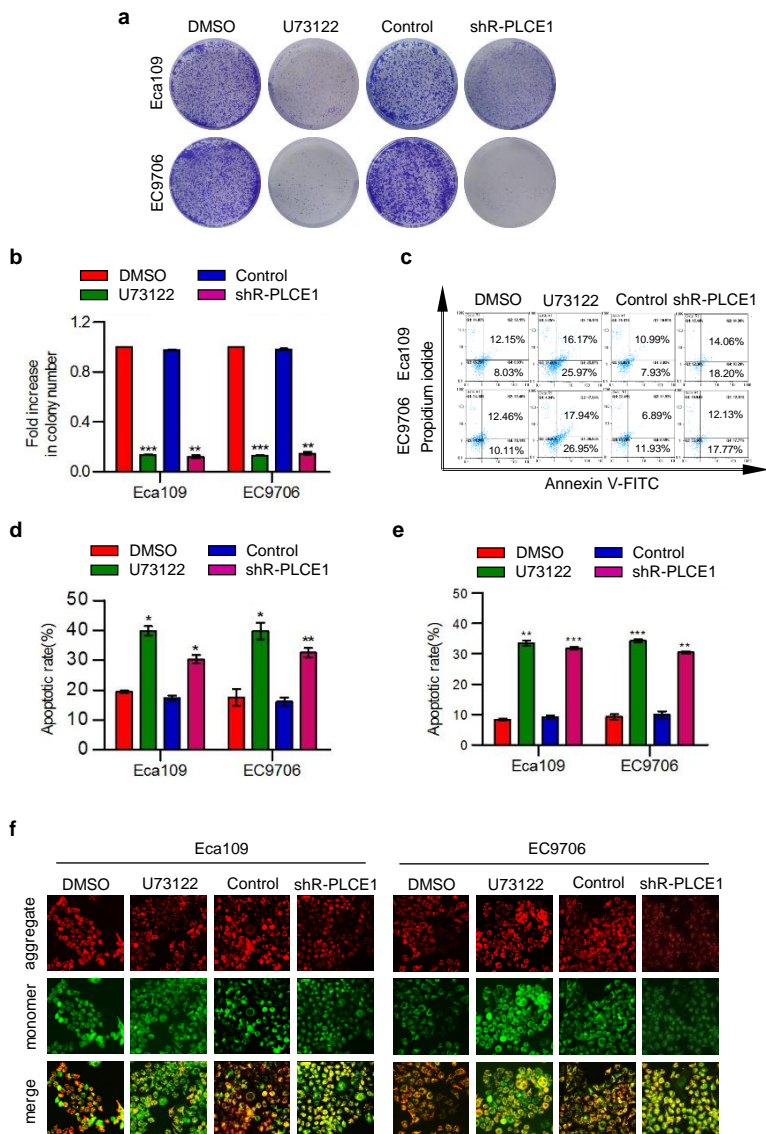

Supplement: Supplementary file 2 — Figure S1. PLCE1 promotes proliferation of esophageal carcinoma cell lines. a Colony formation of ESCC cells decreased by shR-PLCE1 or U73122 treatment and b are means ± SD from three separate experiments. c AV-FITC–PI staining of cells treated as indicated d means ± SD from three independent experiments. e quantification of TUNEL-positive cells. *P < 0.05, **P < 0.01, ***P < 0.001. f Eca109 and EC9706 cells were treated as indicated and JC-1 staining assay. (PDF 363 kb) [file 12943_2018_930_MOESM2_ESM.pdf]

Fig. S2

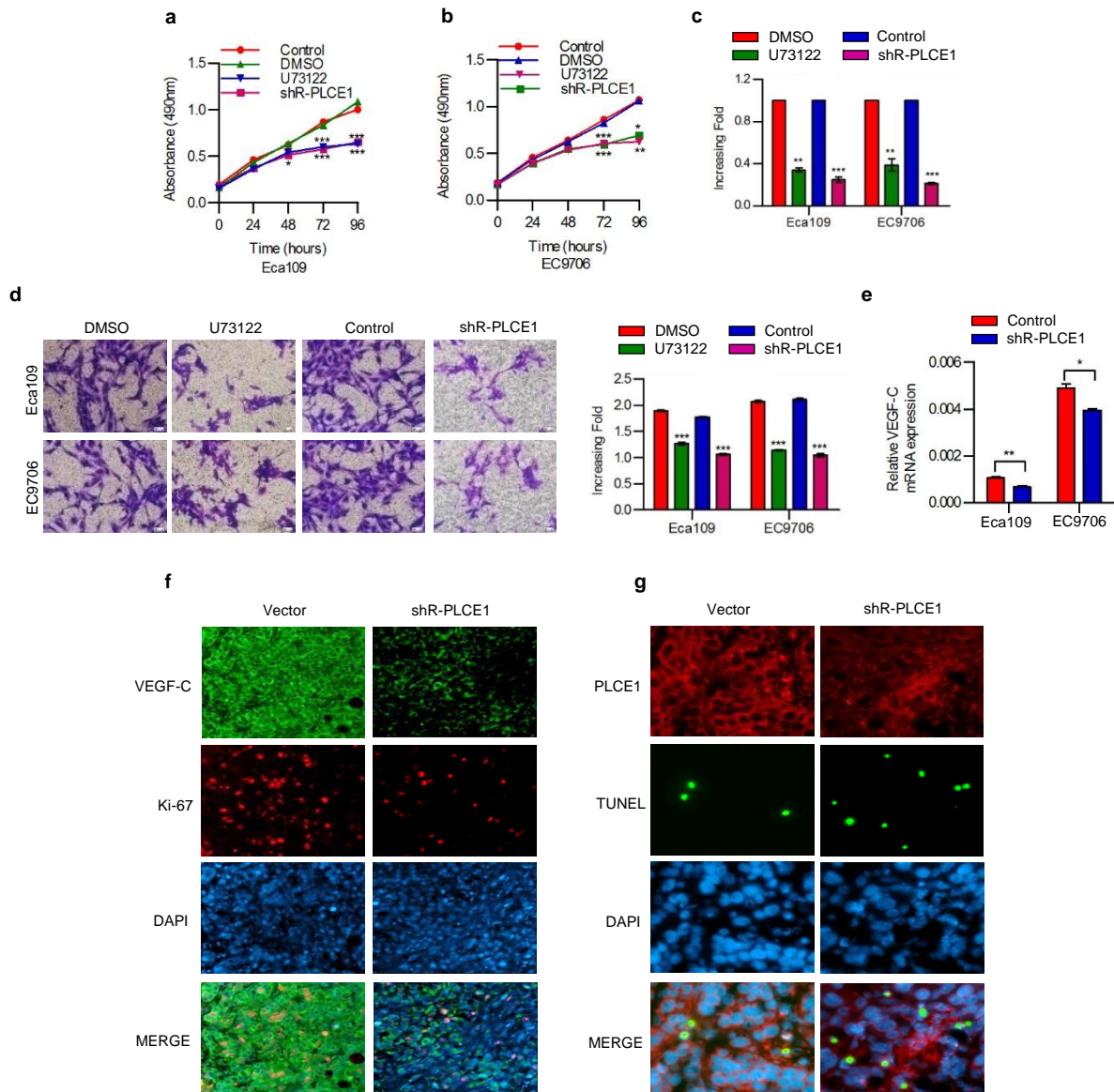

Supplement: Supplementary file 3 — Figure S2. PLCE1 promotes angiogenesis in ESCC cells in vitro and induces aggressiveness in vivo. a, b MTT assay after stimulation with conditioned medium from indicated cells. c Quantification of generated tubes. d Representative images (left panel) and quantification (right panel) of cell invasion by indicated cells in transwell matrix penetration assay. Each bar represents mean ± SD of three independent experiments. **P < 0.01, ***P < 0.001. e Effects of shR-PLCE1 on VEGF-C mRNA expression as detected by real-time PCR analysis. f, g IF show that PLCE1 promotes resistance to apoptosis and angiogenesis in vivo. All data are presented as mean ± SD. *P < 0.05, ***P < 0.001. (PDF 273 kb) [file 12943_2018_930_MOESM3_ESM.pdf]

**Fig. S3**

**a**

|           |   |   |   |   |   |
|-----------|---|---|---|---|---|
| control   | - | - | - | + | - |
| ShR-PLCE1 | - | + | + | - | + |
| TPA       | - | - | + | - | - |
| BIM       | - | - | - | + | + |

Eca109

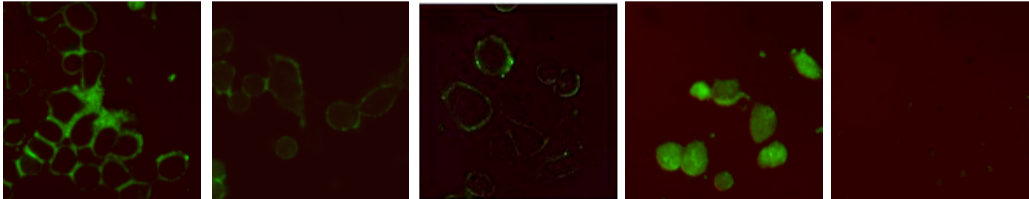

EC9706

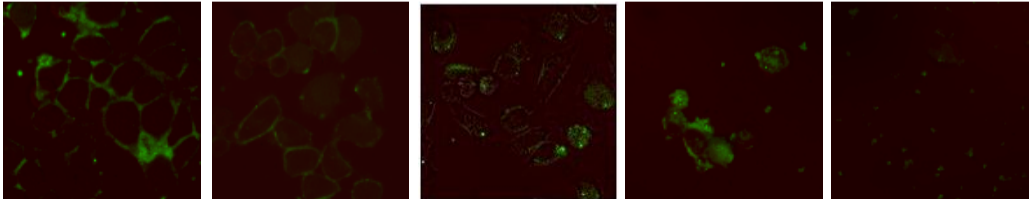

Supplement: Supplementary file 4 — Figure S3. PLCE1 ablation reduces the IP3-dependent intracellular calcium release. a Laser scanning confocal microscopy was used to measure intracellular calcium fluorescence pixel values of ESCC cells after treatment with PLCE1 shRNA, TPA, and BIM. (PDF 95 kb) [file 12943_2018_930_MOESM4_ESM.pdf]

Fig. S4

**a**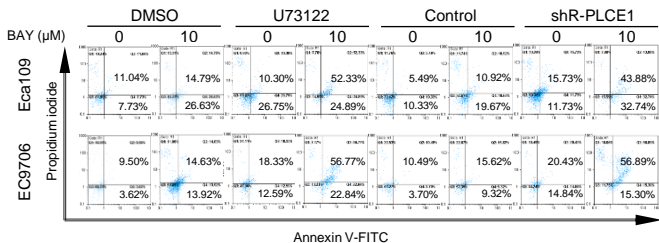**b**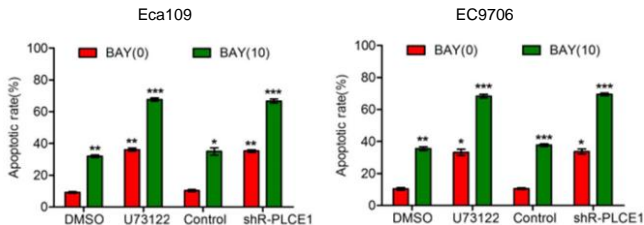**c**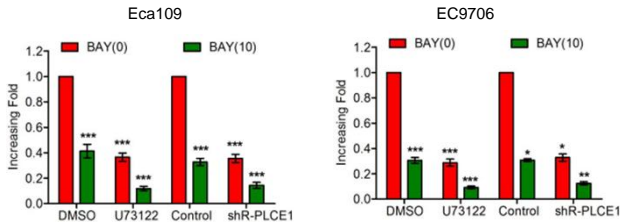

Supplement: Supplementary file 5 — Figure S4. PLCE1 inhibits apoptosis and enhances angiogenesis via activation of the NF-κB signaling pathway in ESCC in vitro. a FITC–PI staining of cells treated with shR-PLCE1 or U73122 or/and Bay11–7082 and results means ± SD from three independent experiments. b quantification of TUNEL-positive cells. *P < 0.05, **P < 0.01, ***P < 0.001. c Tube formation in cells quantification of generated tubes. (PDF 156 kb) [file 12943_2018_930_MOESM5_ESM.pdf]
